# HPV Vaccination and Cervical Cancer Screening: Assessing Awareness, Attitudes, and Adherence in Detained Women

**DOI:** 10.3390/vaccines10081280

**Published:** 2022-08-08

**Authors:** Gabriella Di Giuseppe, Lucio Folcarelli, Raffaele Lanzano, Francesco Napolitano, Maria Pavia

**Affiliations:** Department of Experimental Medicine, University of Campania “Luigi Vanvitelli”, Via Luciano Armanni 5, 80138 Naples, Italy

**Keywords:** adherence, cervical cancer screening, detained women, HPV vaccination, Italy, survey

## Abstract

Background: This study assessed awareness, attitudes, and uptake of human papillomavirus (HPV) vaccination and cervical cancer screening in detained women. Methods: The cross-sectional study was conducted from April to June 2022 in four women prisons in Italy. Results: 41.1% of participants recognized HPV infection as an sexually transmitted diseases (STD), 36.4% identified cervical, and 16.8% oral cancer as an HPV-associated disease. Overall, 70% had never heard of HPV vaccination, and 45.8% believed it is effective to prevent cervical cancer. Among the age-eligible women for HPV vaccination, none reported to have undergone it, nor had talked about it with a physician in the previous year. Only 13.5% declared to have ever undergone cervical cancer screening, and adherence was significantly higher in those who were involved in a working activity in prison, who were aware that HPV infection is an STD and that can cause cervical and oral cancer, and who were older at their first sexual intercourse. Conclusion: These findings documented an extremely low awareness of HPV infection and an unsatisfactory adherence to prevention through HPV vaccination and cervical cancer screening. There is a need for evidence-based interventions for incarcerated women to promote participation in HPV vaccination and cervical cancer screening programs as routine activities.

## 1. Introduction

Infections with human papillomavirus (HPV) and associated diseases including oropharyngeal, anal, cervical, vaginal, vulvar, and penile cancers, are a public health concern worldwide. Nonetheless, the risk of HPV infection and the burden of associated diseases are influenced by geographic, socioeconomic, cultural, and viral-specific factors, as well as by subjective characteristics, such as age, gender, and health status [1]. Incarcerated women show a high occurrence of many of the behaviors which have been correlated to a high risk of HPV infection and the associated diseases, such as multiple sexual partners, sexually transmitted diseases (STD), unprotected sex, and cigarette smoking [2], and a recent meta-analysis has documented in this population a higher prevalence of HPV infection ranging from 10.5% to 55.4%, as well as a higher prevalence of cervical intraepithelial neoplasia (CIN) and cervical cancer [3].

Prevention of HPV infection may rely on primary prevention through vaccination, which has demonstrated to be effective also against CIN and invasive cervical cancers [4,5], and on secondary prevention through organized screening programs using HPV-DNA test and PAP test [6]. In Italy, HPV vaccination is routinely recommended and actively offered free of charge to girls and boys aged 11 or 12 [7]. Moreover, the cervical cancer screening program in Italy includes a Pap smear every 3 years for women aged 25–64 years or an HPV DNA test every 5 years for women ≥30 years to be followed by a Pap smear in case of a positive test [8].

Detained women often belong to disadvantaged and deprived social groups, who are less likely compared to the general population to have access to healthcare services, including preventive services [9]. The health of incarcerated subjects is on average poorer compared to the general population. Indeed, previous investigations showed that detainees compared to people at liberty are more likely to suffer from psychiatric disorders, to have severe drug problems, and to have cancers and other chronic conditions, such as cardiovascular and respiratory diseases [10,11,12,13,14]. Moreover, detainees are also at a higher risk of contracting infectious diseases than the general population due to the conditions of detention, such as staying in a closed community, with poor nutrition, absence of prevention devices, overcrowding, and poor hygiene [15,16]; therefore, from a public health perspective, incarceration may offer a unique opportunity to reach an underserved population and to counterbalance incarceration’s contribution to health disparities [14].

The World Health Organization (WHO) has set a framework for the assessment of prison health system performance and in the section on health services delivery, there is an extensive area dedicated to preventive services for disease prevention, health protection, and health promotion, where vaccinations and cancer screening provided to incarcerated subjects are included [17]. However, there is evidence that the delivery of healthcare to detained subjects does not adequately perform in the prevention of communicable diseases through vaccinations and incarcerated people are often under-immunized against several vaccine-preventable diseases (VPD) [18,19]. Analogously, the delivery of screening tests for cervical cancer is provided in a non-systematic manner, and the overall pathway for positive women is unsatisfactory [20].

To date, there are no studies that have explored these issues in depth in Italian prisons. It was, therefore, of interest to conduct a study on detained women to assess awareness, attitudes, and behaviors concerning HPV infection and cervical cancer, with specific attention to preventive measures including HPV vaccination and cervical cancer screening.

## 2. Materials and Methods

### 2.1. Study Design and Setting

The present cross-sectional study was part of a larger project developed and conducted by the University of Campania “Luigi Vanvitelli” and the Joint Operational Unit for Health Protection at Prison Institutions focused on the investigation of several health-related issues in the incarcerated population [21,22,23,24].

The study was carried out between April and June 2022 in four prisons hosting women in the geographical area of the Campania region, in the South of Italy.

### 2.2. Population Recruitment and Data Collection

All directors of female prisons received an invitation letter containing the study aims, the research protocol, and a consent request to conduct the study.

After having received the approval, an information sheet was distributed to all incarcerated women and each of them was informed about the study aims, that participation was voluntary, and that all data obtained by the survey were strictly confidential.

The survey was compiled in the cells after informed consent was obtained and no incentives were offered for participation. The sample size was calculated assuming that 80% of women adhere to cervical screening programs in accordance with the literature [25,26], a 95% confidence interval, a margin of error of 5%, and a response rate of 80%. This calculation yielded a minimum sample size of 170 participants.

Overall, 214 detained women participated in the survey, out of 241 invited (88.8% response rate).

### 2.3. Survey Instrument

The study was conducted using a self-administered questionnaire which could be filled out by respondents approximately in ten minutes (Supplementary File S1). The survey instrument was built in the Italian language based on items of questionnaires validated in several studies carried out by our and other working groups in prison and community settings [22,23,24,27,28,29,30].

The questionnaire was composed of five sections focused on several topics, including (1) sociodemographic characteristics of incarcerated women and information about the current detention (age, nationality, numbers of sons/daughters, sexual orientation, education level, employment status before and during detention, number of incarceration(s), living in individual or shared cells); (2) anamnestic characteristics (presence or history of underlying chronic conditions and/or STDs) and lifestyle behaviors (smoking, alcohol intake, having had sexual intercourses and age of first sexual intercourse); (3) knowledge about HPV infection and cervical cancer and related prevention strategies; (4) attitudes, behaviors and experience about HPV infection and cervical cancer and related prevention strategies, including HPV vaccination and cervical cancer screening; (5) having received information about prevention strategies against HPV infection and cervical cancer and need for additional general information about HPV and cervical cancer.

In all sections, information was collected using closed-ended questions and multiple choices answers format; in the fourth section, information about attitudes was collected using a 5-point Likert-type scale with a strongly agree/agree/uncertain/disagree/strongly disagree response format. Furthermore, alcohol-related disorders were investigated using the audit-C scale, a validated and effective questionnaire for predicting hazardous alcohol drinking [31,32,33], as prescribed by Italian legislation [34], in a shortened version incorporating only the first three questions: “How often do you consume alcoholic drinks?”; “On days when you drink, how many alcoholic drinks do you consume on average?”; “How often do you drink six or more glasses of alcohol on a single occasion?” The score for each answer was from 0 to 4, and the final score was from 0 to 12. The test reveals an above-average risk of developing an alcohol-related disorder (consumer at risk, harmful consumption, or alcohol dependence) upon a total score of three points or more.

### 2.4. Statistical Analysis

First, data collected were analyzed by performing a descriptive analysis to explore the characteristics of the population; continuous and categorical variables were described using, respectively, means/standard deviations and absolute values and relative frequencies. Second, bivariate analyses were conducted to evaluate the association between having had a PAP smear for cervical cancer screening and potential determinants by using Student’s *t*-test for continuous variables and the chi-square test for categorical variables. Next, a multivariate stepwise logistic regression analysis was performed to evaluate the potential determinants associated with having had a PAP smear for cervical cancer screening (no = 0; yes = 1). The independent variables that were shown to be associated at the univariate analysis or that were judged to potentially have an influence on the investigated outcome were included in the model. A detailed description of the independent variables included in the model and the related categories is reported in a Supplementary File (Supplementary File S2).

The level of statistical significance was defined by a *p*-value equal to or less than 0.05. Furthermore, adjusted odds ratios (ORs) and 95% confidence intervals (CIs) were calculated. All statistical analyses were performed using STATA software, version 15.1, Stata Corporation, College Station, TX, USA [35].

### 2.5. Pilot Study and Ethical Statement

Prior to the beginning of the survey, the questionnaire was pre-tested on 50 women to ensure correct interpretation, reliability, and feasibility of the questions, no changes were made to the survey instrument, and the results from these women were included in the final sample. The study was approved by the Ethics Committee of the Teaching Hospital of the University of Campania “Luigi Vanvitelli” (prot. 13275/i, 2022).

## 3. Results

### 3.1. Socio-Demographic, Detention, and Anamnestic Characteristics of the Study Population

Detained women reported a mean age of 44.4 years (range 18–77), only 13.1% were not Italians, 91.6% reported a heterosexual orientation, and 86% had at least one son or daughter, 21.9% reported a high school or university degree, and 41.6% declared to have an occupation before detention. For almost two-thirds of the responders (65.4%), this was the first experience of incarceration, with 11.7% reporting to live in individual cells and 43.9% to be involved in working activities in the prison. The frequency of detained women reporting at least one chronic disease was 45.3%, whereas a diagnosis of STD was declared by 7% of participating women. The large majority reported current smoking (81.8%), with 4.6% having started within prison, and for 13.6% an alcohol use disorder was assessed through the audit-C score. Almost all had had sexual intercourse (99.1%), and the mean age at sexual debut was 16.9 years (Supplementary File S3).

### 3.2. Knowledge about HPV Infection, Cervical Cancer, and Related Prevention

Only 41.1% of responding women recognized HPV infection as an STD, with almost half (48.6%) declaring they do not know. Regarding the HPV-associated diseases, only 36.4% identified cervical cancer and 16.8% oral cancer, with most of the sample responding they do not know (55.1% and 65.4%, respectively). Instead, most of the women have heard about the PAP test (85.9%) and 78.5% are aware that HPV infection usually goes away without any treatment. Poor knowledge was observed concerning HPV vaccination, with more than 70% who declare to have never heard about this vaccination, only 39.2% know that HPV vaccination is effective in the prevention of genital warts, and only 45.8% are aware that it is effective to prevent cervical cancer. Furthermore, only 19.2% and 10.8% are aware that it is effective in sexually active women and in those who have already been exposed to HPV, respectively. Finally, the composite variable on knowledge showed that only 11.2% of the women are aware that HPV infection is an STD, and that HPV may cause cervical and oral cancer (Table 1).

### 3.3. Attitudes towards HPV Infection, Cervical Cancer, and Related Prevention

Only 15.9% and 9.3% believe they would get HPV infection throughout life, and that their lifestyle would increase the risk of HPV infection, respectively. The sexual transmission of HPV infection is quoted as easy by 71.5% of participants, and 71% would find it embarrassing to have genital warts. Three-quarters of respondents (75.7%) agree that being diagnosed with cervical cancer would have negative consequences on their lives, and 68.7% are aware that it can cause death. Moreover, 26.7% and 34.1% believe that their risk of HPV infection and of cervical cancer is the same of women who had never been incarcerated, respectively (Table 2).

Regarding vaccinations, 47.7% believe or are uncertain that vaccines in general are more dangerous than safe, 36.9% that the HPV vaccine is safe, while only 9.3% that it may cause serious side effects. Moreover, 80.4% believe or are uncertain that HPV vaccination is not necessary if one gets a PAP test and 46.8% agree that the HPV vaccine could save their lives (Table 2).

### 3.4. Preventive Behavior towards HPV Infection and Cervical Cancer

Among the age-eligible women for HPV vaccination, none reported to have undergone it, nor had talked about it with a physician in the previous year. Most respondents were eligible for cervical cancer screening, but only 13.5% reported to have ever undergone it. Among these women, 46.4% reported to have received a PAP test in prison, 39.3% in the community, and 14.3% in both; additionally, 14.3% declared at least one abnormal PAP test result, and 1.8% a diagnosis of cervical cancer (Table 3).

**Figure 1:**
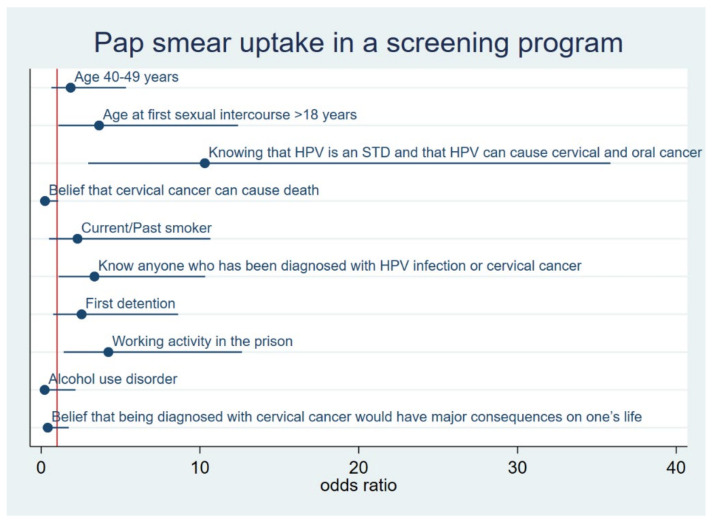
Graph illustrating the results of multiple logistic regression analysis to characterize factors associated with the adherence to cervical cancer screening programs.

The results of the multiple logistic regression showed that women who had ever undergone a PAP smear in a screening program had significantly higher odds of having a familiar or friend history of HPV infection or cervical cancer (OR = 3.358, 95% CI = 1.091–10.331), of being involved in a working activity in prison (OR = 4.233, 95% CI = 1.417–12.648), of being aware that HPV infection is an STD and that can cause cervical and oral cancer (OR = 10.305, 95% CI = 2.962–35.851), and of being older at their first sexual intercourse (OR = 3.645, 95% CI = 1.071–12.397) (Table 4 and Figure 1).

### 3.5. Sources of Information

Only 5.1% reported to have been informed about HPV infection and cervical cancer prevention strategies during permanence in prison, and 93% declared to have a need for additional information on these topics.

## 4. Discussions

Detained subjects are an understudied population, and, among them, women are even less investigated. To our knowledge, this is one of the few studies assessing the awareness of detained women on the risks correlated to HPV infection, with specific attention to the prevention of cervical cancer through vaccination and screening tests, as well as participation in HPV infection and cervical cancer preventive activities.

### 4.1. Knowledge and Attitudes about HPV Infection, Cervical Cancer, and Related Prevention

The results of this survey have revealed very poor knowledge of HPV infection and associated diseases, along with limited awareness of the role of HPV vaccination, whereas an expected higher knowledge, although unsatisfactory, was revealed on the role of the PAP test, probably related to the less recent introduction of this procedure as compared to HPV vaccination. These findings are even less satisfactory than those reported by Moore et al. in detained women in the USA, as regards to knowledge of the association of HPV infection with cervical cancer (57% vs. 36.4% in this study), with oral cancer (19% vs. 16.8%), as well as on awareness that HPV infection is an STD (61% vs. 41.1%) [36]; moreover, knowledge on the availability of HPV vaccination, reported by 26.1% of respondents, was similar to that described by Pankey et al. (22%) and lower than that reported by Allison et al. (70%), both investigating detained women in the USA [27,37]. These findings are not surprising, since several studies have documented scarce reproductive health literacy and misconceptions on the role of HPV in the development of cervical cancer [25,38,39] in detained women.

Consistently with knowledge, more concern is related to statements involving cervical cancer, whereas lower interest is devoted to HPV infection and vaccination. Overall, there is a low perception of the risk of HPV infection and associated health consequences, and specifically on the potential of lifestyle-related behaviors, although the sexual transmission of HPV is reported to be easy by the majority of women. Moreover, the finding that almost half of respondents expressed concerns about the safety of vaccinations is alarming and mirrors perceptions on HPV vaccination, since only one-fifth of women disagreed with the statement that it can cause serious side effects.

### 4.2. Preventive Behavior towards HPV Infection and Cervical Cancer

The findings of this study demonstrate that in detained women the availability of preventive measures against HPV infection and cervical cancer still represents a missed opportunity even in the context of a universal healthcare coverage system; indeed, it is unacceptable that none of the eligible women had undergone HPV vaccination and only 13.5% had ever had a PAP smear in a screening program. It has been described that detained women report less access to preventive activities in the community [26], but the results of the present study have shown significantly lower adherence compared to data reported in most of the existing literature. A recent systematic review investigating cervical cancer screening in correctional facilities [40] has documented a higher prevalence of PAP smear in the previous 3 years ranging from 77.2% [25] to 84% [41], and in most cases, these figures were lower compared to those reported in the general population. Moreover, these results show a lower adherence to cervical cancer screening also in comparison with the general population and with immigrant women of the same area, where 85.4% of women of the general population (24.6% within an organized screening program) [29] and 39.1% among immigrant women [42], reported to have ever undergone a cervical cancer screening test. This low participation in organized screening programs is concerning, since these programs are more likely to be attended by socio-economically disadvantaged women, and low coverage in these programs is associated with health and social inequalities [43]. One striking result of this study, however, is that among women who have undergone cervical cancer screening, the more numerous group has been involved in this procedure inside the prison, demonstrating that incarceration may become an opportunity for access to preventive services for this underprivileged population [14].

An even more worrisome result is provided by the absence of HPV-vaccinated women among those who were eligible, coupled with the reporting that none of the eligible women had been informed about the vaccination by a prison physician in the previous year. There is a dearth of information on HPV vaccination in incarcerated women, with only two studies reporting 34% [36] and 35% [27] coverage in this population. HPV coverage in Italy is still unsatisfactory in the general population; a study conducted in the same area reported 40.5% HPV vaccination coverage in 18–30-year-old women [30], whereas data from the Ministry of Health updated to December 2020 show HPV vaccination coverage ranging from 46.6% in the 1996 birth cohort to 75.5% in the 2005 birth cohort for at least one dose [44].

A low prevalence of HPV vaccination and of cervical cancer screening was expected in this population, but the results of this survey are far below most of the findings reported in the literature and stimulate the implementation of preventive interventions targeted at incarcerated women to promote HPV vaccination and cervical cancer screening, thus catching the opportunity within prison to increase the adherence to these preventive measures in a population which is hard to reach in the community. Assessing HPV vaccination and cervical cancer screening status in clinical records at entry may then address women to dedicated health promotion activities to understand the indications and benefits of HPV vaccination and cervical cancer screening, followed by promotion of access to these preventive activities. Indeed, studies that have implemented targeted health promotion activities in this population have been successful [38], and those which have explored the willingness of incarcerated women to undergo HPV vaccination [27,36] and cervical cancer screening [45,46,47,48] have generally found a positive attitude towards adhesion to these programs if offered in prison.

One of the main objectives of the study was to evaluate the predictors of PAP smear uptake among the surveyed population. According to previous investigations on incarcerated women, sociodemographic and detention characteristics were not found to be significantly associated with PAP smear uptake [46,49], whereas a higher uptake was found to be associated with older age and longer lengths of incarceration in some studies [50,51,52,53]. In this study, being aware that HPV infection is an STD that can cause cervical and oral cancer and having a familiar or friend history of HPV infection or cervical cancer were among the stronger predictors. The role of knowledge of HPV infection and personal experience of cancer was confirmed as a determinant of preventive measures uptake in previous investigations conducted in the US and Peru [37,52]. Therefore, according to the results on determinants, in order to enhance adherence to cervical cancer screening programs in this population, there is a need for increasing awareness of the risks correlated to HPV infection and of the availability of preventive measures, as well as promoting self-confidence and empowerment of detained women in the ability to take action to improve their health.

### 4.3. Public Health Implications of the Study

The results of this study showed that the adherence to preventive measures against HPV infection and cervical cancer among incarcerated women is worryingly inadequate. Therefore, there is plenty of room for health policymakers and those responsible for providing care to women deprived of liberty to more effectively implement prevention strategies within the prison. This would fulfill the healthcare gap between incarcerated women and citizens at liberty, which is responsible for inequity in health even in a country where health is a constitutional right. Therefore, detention conditions in prisons represent an emergency in terms of rights and public health and there is an urgent need got more efforts to counter this emergency by governments, and social and healthcare services.

### 4.4. Limitations of the Study

The findings of this study should be examined taking into account limitations that may influence the interpretation of the results. First, this study adopted a cross-sectional design, and thus it allows only to determine associations, but not causality between determinants and outcomes. Second, the survey relied on self-reporting and there was no objective verification through chart review as regards HPV vaccination or cervical cancer screening adherence. Third, the sample of incarcerated women is small, and results on determinants may have been affected by low power, reducing our ability to find associations between determinants and outcomes. However, the population of detained women is small compared to detained males and recruitment involved the majority of prisons in our area, and the study has provided novel information on a poorly investigated population. Moreover, the large participation with a very high response rate reduces the risk of selection bias and allows generalizability of results to the southern Italy population of detained women. Future research involving a larger geographic sample would be of interest.

## 5. Conclusions

The results of the study have documented an extremely low awareness of the risks correlated to HPV infection and an absolutely unsatisfactory adherence of incarcerated women to prevention through HPV vaccination and cervical cancer screening. Future research focusing on evidence-based interventions to incorporate and promote participation in HPV vaccination and cervical cancer screening programs as routine activities offered to incarcerated women would be valuable for this population to reduce disparities of access to preventive services in the community.

## Figures and Tables

**Table 1:** Knowledge about HPV infection and cervical cancer.

Knowledge Items	Yes	Do Not Know	No
	*n* (%)	*n* (%)	*n* (%)
HPV is a sexually transmitted disease (STD)	**88 (41.1)**	104 (48.6)	22 (10.3)
HPV can cause:			
Abnormal PAP smears	**85 (39.7)**	117 (54.7)	12 (5.6)
Cervical cancer	**78 (36.4)**	118 (55.1)	18 (8.5)
Genital warts	**63 (29.4)**	134 (62.6)	17 (8)
Bladder infection	66 (30.8)	131 (61.2)	**17 (8)**
Skin cancer	19 (8.9)	147 (68.7)	**48 (22.4)**
Oral cancer	**36 (16.8)**	140 (65.4)	38 (17.8)
HPV vaccination is effective at preventing genital warts	**84 (39.2)**	110 (51.4)	20 (9.4)
HPV vaccination is effective in preventing cervical cancer	**98 (45.8)**	100 (46.7)	16 (7.5)
HPV vaccination prevents other STDs such as HIV, chlamydia, and others	28 (13.1)	153 (71.5)	**33 (15.4)**
HPV vaccination is effective in women who are already sexually active	**41 (19.2)**	153 (71.5)	20 (9.3)
HPV vaccination is effective in women who have already been exposed to HPV	**23 (10.8)**	161 (75.2)	30 (14)
HPV infection usually goes away in its own	**168 (78.5)**	45 (21.1)	1 (0.4)

Number and percentages referring to correct answers are in bold.

**Table 2:** Attitudes and beliefs towards HPV infection, cervical cancer, and related preventive measures.

	Strongly Agree or Agree	Uncertain	Strongly Disagree or Disagree
Items	*n* (%)	*n* (%)	*n* (%)
I will get HPV infection during my life	34 (15.9)	62 (28.9)	118 (55.2)
My lifestyle increases the risk to get HPV infection	20 (9.3)	56 (26.1)	138 (64.6)
I have the same risk of HPV infection as women who have never been incarcerated	57 (26.7)	79 (36.9)	78 (36.4)
I have the same risk of cervical cancer as women who have never been incarcerated	73 (34.1)	77 (36)	64 (29.9)
HPV infection transmission is easy through sexual partners	153 (71.5)	50 (23.4)	11 (5.1)
It is embarrassing to have genital warts	152 (71)	54 (25.3)	8 (3.7)
Being diagnosed with cervical cancer would have major negative consequences on my life	162 (75.7)	45 (21)	7 (3.3)
Cervical cancer can cause death	147 (68.7)	56 (26.1)	11 (5.2)
Generally, vaccines are more dangerous than safe	50 (23.4)	52 (24.3)	112 (52.3)
HPV vaccination is safe	79 (36.9)	112 (52.3)	23 (10.8)
HPV vaccination can cause serious side effects	20 (9.3)	153 (71.5)	41 (19.2)
HPV vaccination is not necessary if I get regular PAP test	73 (34.1)	99 (46.2)	42 (19.7)
HPV vaccination can save my life *	36 (46.8)	28 (36.3)	13 (16.9)

* Only in age-eligible for HPV vaccination (aged ≤26 years from 2007 to the present—77 observations).

**Table 3:** Behaviors towards HPV infection and cervical cancer prevention and treatment.

Items	*n* (%)
Have you ever got HPV vaccination? ^1^	
Yes	0 (0)
No	77 (100)
Have you ever received a diagnosis of HPV infection?	
Yes	8 (3.3)
No	206 (96.7)
Have you ever had a PAP smear included in a screening program? ^2^	
Yes	28 (13.5)
No	173 (83.2)
Do not know	7 (3.3)
Where did you take a PAP smear? ^3^	
In the community, before incarceration	11 (39.3)
In prison	13 (46.4)
Both	4 (14.3)
Have you ever had an abnormal PAP smear? ^3^	
Yes	4 (14.3)
No	23 (82.1)
Do not know	1 (3.6)
After the abnormal PAP smear test, what did you do? ^4^	
Colposcopy/biopsy	3 (75)
I took another PAP smear	1 (25)
Have you ever had a diagnosis of cervical cancer?	
Yes	4 (1.8)
No	210 (98.2)
Did you follow (are you following) a specific treatment for the cervical cancer? ^5^	
Surgery	3 (75)
Chemotherapy and radiotherapy	1 (25)

^1^ Only in age-eligible for HPV vaccination women (aged <26 years from 2007 to the present—77 observations), ^2^ only for age-eligible women (aged ≥25 years—208 observations), ^3^ only for those who answered “Yes” at “Have you ever had a PAP smear test included in a screening program?”, ^4^ only for those who answered “Yes” at the question “Have you ever have an abnormal PAP smear test?”, ^5^ only for those who answered “Yes” at the question “Have you ever had a diagnosis of cervical cancer?”.

**Table 4:** Multiple logistic regression analysis to characterize factors associated with the adherence to cervical cancer screening programs.

^ Model. Pap Smear Uptake in a Screening Program				Total	Pap Smear Uptake
	OR	95% CI ^°^	*p*	*n*	*n* (%)
Age, years					
25–39	1 *			65	6 (9.2)
40–49	1.855	0.644–5.341	0.252	70	12 (17.1)
≥50	Backward elimination			66	8 (12.1)
First detention					
No	1 *			67	8 (11.9)
Yes	2.546	0.751–8.629	0.133	134	20 (14.9)
Working activity in the prison					
No	1 *			114	9 (7.9%)
Yes	4.233	1.417–12.648	**0.010**	87	19 (21.8)
Smoking habit					
Never smoker	1 *			33	4 (12.1)
Current/past smoker	2.288	0.491–10.655	0.291	168	24 (14.3)
Alcohol use disorder					
No	1 *			176	26 (14.8)
Yes	0.218	0.022–2.160	0.193	25	2 (8)
Age at first sexual intercourse, years					
≤15	1 *			66	8 (12.1)
16–18	Backward elimination			96	10 (10.4)
≥19	3.645	1.071–12.397	**0.038**	39	8 (20.5)
Know anyone who has been diagnosed with HPV infection or cervical cancer					
No	1 *			153	16 (10.4)
Yes	3.358	1.091–10.331	**0.035**	48	12 (25)
Knowing that HPV is an STD and that HPV can cause cervical and oral cancer					
No	1 *			177	19 (10.7)
Yes	10.305	2.962–35.851	**<0.001**	24	9 (37.5)
Belief that being diagnosed with cervical cancer would have major consequences on one’s life					
No	1 *			50	13 (26)
Yes	0.413	0.098–1.734	0.228	151	15 (9.9)
Belief that cervical cancer can cause death					
No	1 *			62	16 (25.8)
Yes	0.239	0.053–1.070	0.061	139	12 (8.6)

* Reference category; ° confidence interval. ^ The following variables were removed from the model by the backward elimination procedure: education level, having sons/daughters, working activity before detention, ever been diagnosed with an STD, belief that one’s lifestyle increases the risk of HPV infection, having received information about prevention strategies against HPV infection and cervical cancer during detention, the need for additional information about HPV infection, HPV vaccination, and cervical cancer.

## Data Availability

The data that support the findings of this study are available from the corresponding author, [M.P.], upon reasonable request.

## Supplementary Materials

The following supporting information can be downloaded at: https://www.mdpi.com/article/10.3390/vaccines10081280/s1, S1: Questionnaire; S2: Description of the independent variables included in the model and the related categories; S3: Table of the characteristics of the study population.
