# Supplementary material for: HPV Vaccination and Cervical Cancer Screening: Assessing Awareness, Attitudes, and Adherence in Detained Women
Source: Vaccines (Basel). 2022 Aug 8;10(8):1280. doi: 10.3390/vaccines10081280 (PMC9416201; doi:10.3390/vaccines10081280)
Supplement: Supplementary file 1 [file vaccines-10-01280-s001.zip › Supplementary File S1.pdf]

QUESTIONNAIRE

MARK WITH AN X OR, WHEN INDICATED, REPLY BRIEFLY

A. SOCIO-DEMOGRAPHIC AND DETENTION CHARACTERISTICS

A.1. Year of birth____________ **A.2.** Nationality ________________

A.3. Do you have sons/daughters? □No □Yes, how many? _________________

A.4. Sexual orientation: □ Heterosexual □ Lesbian □ Bisexual □ Other ________

A.5. What is the highest education leaving certificate, diploma or education degree you have obtained?

□ None □ Primary school □ Middle school □ High school □ University degree □ Other __________

A.6. Before this detention, what was your work activity? ____

A.7. Is this your first detention?

□ Yes □ No I have already spent in prison______ years, and _____months

A.8. Do you have a work activity in prison? □ No □ Yes

A.9. Type of prison cell: □ Individual □ Shared

B. ANAMNESTIC CHARACTERISTICS

B.1. Do you have any chronic disease? (e.g., diabetes, high blood pressure, etc.)?

□ No □ Yes, specify **(more than one disease allowed)** ____

B.2. Have you smoked at least 100 cigarettes in your life (One hundred cigarettes is equal to 5 packs)? □ No (go to **B7**) □ Yes

B.3. How old were you when you started smoking cigarettes? _______, years

B.4. Do you now smoke cigarettes? □No (go to **B6**) □Yes

B.5. How many cigarettes do you smoke per day? ___________

B.6. Did you smoke before entering prison? □No □Yes

B.7. How often do you consume alcoholic drinks?

□Never (go to **B10**) □Monthly or less □ 2 to 4 times a month □ 2 to 3 times a week □ 4 or more times a week

B.8. On days when you drink, how many alcoholic drinks do you consume on average?

□ 1 o 2 □ 3 o 4 □ 5 o 6 □ 7 o 9 □ 10 or more

B.9. How often do you drink six or more glasses of alcohol on a single occasion?

□ Never □ Less than monthly □ Monthly □ Weekly □ Daily or almost daily

B.10. Have you ever had a sexual intercourse?

□ No (go to **section C**) □ Yes, age of first sexual intercourse: ________

B.11. Have you ever received a diagnosis of a Sexually Transmitted Disease (STD)?

□No □Yes, specify **(more than one disease allowed)** _________________

C. KNOWLEDGE ON HPV INFECTION AND CERVICAL CANCER PREVENTION

C.1. Is HPV infection a STD? □No □Yes □Do not know

C.2. Have you ever heard about PAP smear? □No □Yes

C.3. For each statement answer "Yes", "No", or "I do not know".

HPV infection can cause:

	**Yes**	**No**	**I do not know**
Abnormal PAP smears	□	□	□
Genital warts	□	□	□
Bladder infection	□	□	□
Cervical cancer	□	□	□
Skin cancer	□	□	□
Oral cancer	□	□	□

C.4. Does HPV infection require medical treatment or does it usually go away without any treatment?

□ Usually goes away without any treatment □Requires medical treatment □Do not know

C.5. Do you know anyone who has been diagnosed with HPV infection? □No □Yes

C.6. Do you know anyone who has been diagnosed with cervical cancer? □No □Yes

C.7. Have you ever heard about HPV vaccination? □No □Yes

C.8. Does HPV vaccine prevent other STDs (eg. HIV, Chlamydia, etc.)? □Yes □No □Do not know

C.9. Is HPV vaccine effective in sexually active women? □Yes □No □Do not know

C.10. Is HPV vaccine effective in those who have already been exposed to HPV infection? □Yes □No □Do not know

D. ATTITUDES AND BEHAVIOURS TOWARD HPV INFECTION AND CERVICAL CANCER PREVENTION

D.1. This section presents a series of statements regarding HPV infection and cervical cancer. For each statement answer whether you strongly agree, agree, are uncertain, disagree, or strongly disagree

	Strongly agree	Agree	Uncertain	Disagree	Strongly disagree
I will get HPV infection during my life	□	□	□	□	□
My lifestyle increases the risk to get HPV infection	□	□	□	□	□
I have the same risk of HPV infection as women who have never been incarcerated	□	□	□	□	□
I have the same risk of cervical cancer as women who have never been incarcerated	□	□	□	□	□
HPV infection transmission is easy through sexual partners	□	□	□	□	□
It is embarrassing to have genital warts	□	□	□	□	□
Being diagnosed with cervical cancer would have major negative consequences on my life	□	□	□	□	□
Cervical cancer can cause death	□	□	□	□	□

D.2. This section presents a series of statements regarding HPV vaccination. For each statement answer whether you strongly agree, agree, are uncertain, disagree, or strongly disagree

	Strongly agree	Agree	Uncertain	Disagree	Strongly disagree
Generally, vaccines are more dangerous than safe	□	□	□	□	□
HPV vaccination is safe	□	□	□	□	□
HPV vaccination can cause serious side effects	□	□	□	□	□
HPV vaccination is effective in preventing genital warts	□	□	□	□	□
HPV vaccination is effective in preventing cervical cancer	□	□	□	□	□
HPV vaccination is not necessary if I get regular PAP test	□	□	□	□	□
HPV vaccination can save my life **(answer only if you are not older than 40 years)**	□	□	□	□	□

D.3. In the previous year, have you talked with a physician about HPV vaccination? **(answer only if you are not older than 26 years)**
□No □Yes, while incarcerated □Yes, while in community □ Do not know □ I have not seen a physician in the last year
D.4. Have you been vaccinated against HPV?**(answer only if you are not older than 40 years)** □ No □Yes □Do not know
D.5. Have you ever received a diagnosis of HPV infection? □No □Yes, when? (Year of diagnosis) _______________
D.6. Have you ever had a PAP smear included in a screening program? **(answer only if you are aged ≥ 25 years)**
□No (go to **D10**) □Yes □Do not know
D.7. Where did you take a PAP smear? **(more than one disease allowed)**
□ in the community, before incarceration □ in prison
D.8. Have you ever had an abnormal PAP smear? □No (go to **D10**) □Yes □Do not know
D.9. After the abnormal PAP smear test, what did you do?
□Colposcopy/Biopsy □I took another PAP smear □Nothing □Do not know
D.10. Have you ever had a diagnosis of cervical cancer? □No (go to section **E**) □Yes
D.11. Did you follow (are you following) a specific treatment for the cervical cancer?
□No □Yes, which treatment? → □Surgery □Chemotherapy □Radiotherapy

SOURCES OF INFORMATION ON HPV INFECTION AND CERVICAL CANCER PREVENTION

E.1. Since you are in prison, have you received information about prevention strategies against HPV infection and cervical cancer?
□No □Yes
E.2. Do you feel you need additional information on HPV infection, cervical cancer and, in general, about health topics? □No □Yes
