# Supplementary material for: HPV Vaccination and Cervical Cancer Screening: Assessing Awareness, Attitudes, and Adherence in Detained Women
Source: Vaccines (Basel). 2022 Aug 8;10(8):1280. doi: 10.3390/vaccines10081280 (PMC9416201; doi:10.3390/vaccines10081280)
Supplement: Supplementary file 1 [file vaccines-10-01280-s001.zip › Supplementary File S2.pdf]

Variables included in the logistic regression model with related categories

Model. Pap smear uptake in a screening program

**Independent variables**	**Code**
Age, in years	25-39=1
	40-49=2
	≥50=3
Nationality	Foreigners=0
	Italians=1
Marital status	Unmarried/widowed/separated/divorced=0
	Married/cohabitant=1
Having sons/daughters	No=0
	1=1
	>1=2
	Yes=1
Education level	None/primary school/middle school=0
	High school or university degree=1
Working activity before detention	Unemployed=0
	Employed=1
First detention	No=0
	Yes=1
Working activity in the prison	No=0
	Yes=1
Presence of chronic disease(s)	No=0
	Yes=1
Smoking habit	Never smoker=0
	Current/past smoker=1
Presence of alcohol use disorder	No=0
	Yes=1
Age at first sexual intercourse, in years	≤15=1
	16-18=2
	≥19=3
Ever been diagnosed an STD	No=0
	Yes=1
Know anyone who has been diagnosed with HPV infection or cervical cancer	No=0
	Yes=1
Knowing that HPV is an STD and that HPV can cause cervical and oral cancer	No=0
	Yes=1
Belief that one's lifestyle increases the risk of HPV infection	Uncertain/disagree/strongly disagree=0
	Strongly agree/agree=1
Belief that being diagnosed with cervical cancer would have major negative consequences on one's life	Uncertain/disagree/strongly disagree=0
	Strongly agree/agree=1
Belief that cervical cancer can cause death	Uncertain/disagree/strongly disagree=0
	Strongly agree/agree=1

Having received information about prevention strategies against HPV infection and cervical cancer during detention	No=0
	Yes=1
Need of additional information about HPV infection, HPV vaccination, and cervical cancer	No=0
	Yes=1
