# Supplementary material for: HPV Vaccination and Cervical Cancer Screening: Assessing Awareness, Attitudes, and Adherence in Detained Women
Source: Vaccines (Basel). 2022 Aug 8;10(8):1280. doi: 10.3390/vaccines10081280 (PMC9416201; doi:10.3390/vaccines10081280)
Supplement: Supplementary file 1 [file vaccines-10-01280-s001.zip › Supplementary File S3.pdf]

Table S1. Characteristics of the study population (n=214).

**Characteristics**		
**Socio-Demographics**	**n**	**%**
Age, years	44.4 ± 10.3 (18-77)*	
18-39	74	34.6
40-49	73	34.1
50-77	67	31.3
Nationality		
Italians	186	86.9
Foreigners	28	13.1
Sons/daughters		
Yes	184	86
No	30	14
Sexual orientation		
Heterosexual	196	91.6
Homosexual/ Bisexual	18	8.4
Education level		
None/primary school	62	29
Middle school	105	49.1
High school or university degree	47	21.9
Occupation before detention		
Unemployed	125	58.4
Employed	89	41.6
**Detention**		
First detention		
Yes	140	65.4
No	74	34.6
Length of detention, months	58.4 ± 72.2 (1-360)*	
Working activity in the prison		
Yes	94	43.9
No	120	56.1
Type of cell		
Individual	25	11.7
Shared	189	88.3
**Anamnestic**		
At least one chronic disease		
No	117	54.7
Yes	97	45.3
Cardiovascular diseases[a]	30	30.9
Endocrine and metabolic diseases[a]	19	19.5
Respiratory diseases[a]	13	13.4
Psychiatric diseases[a]	13	13.4
Gastrointestinal diseases[a]	8	8.2
Genitourinary diseases[a]	6	6.1
Rheumatic diseases[a]	6	6.1
Other[a]	13	13.4
Smoking habit		
Current smoker	175	81.8
Past smoker	5	2.3
Never smoker	34	15.9
Number of cigarettes smoked per day[b,c]	20 (15-30)**	

Age at starting smoking[b,c]	15.6 ± 6.7 (6-48)*	
Alcohol use disorder[d]		
No (< 3)	185	86.4
Yes (≥ 3)	29	13.6
Ever been diagnosed an STD[e]		
No	199	93
Yes	15	7
HPV[a]	7	46.7
HIV[a]	5	33.3
Syphilis[a]	2	13.3
Hepatitis B[a]	1	6.7
Know anyone who has been diagnosed with HPV infection		
Yes	24	11.2
No	190	88.8
Know anyone who has been diagnosed with cervical cancer		
Yes	37	17.3
No	177	82.7

* Mean ± Standard deviation (range). ** Median value and interquartile range (IQR). [a] Multiple responses allowed. [b] Among current and past smokers. [c] Not including e-cigarettes, cigars, etc. [d] Calculated through the AUDIT-C test: a score of 3 or more is considered a positive screen for alcohol misuse. [e] Sexually transmitted disease (STD).
